# Optimising Mental Health Care for People With Neurological Conditions in the UK: Evidence-Based Models for Integrated Neuropsychiatric Services

**DOI:** 10.7759/cureus.102546

**Published:** 2026-01-29

**Authors:** Naireen Asim, Al Mahdin Ornob Miah

**Affiliations:** 1 Geriatrics, Surrey and Sussex Healthcare Trust, London, GBR; 2 Hospital Medicine, Barts Health Trust, London, GBR

**Keywords:** health service models, inpatient services, integrated care, liaison psychiatry, mental health services, multidisciplinary care, neurological disorders, neuropsychiatry, psychiatric comorbidity, united kingdom

## Abstract

Neuropsychiatry bridges neurology and psychiatry by addressing mental health symptoms arising from brain disorders. Psychiatric comorbidities are common in neurological conditions, yet access to appropriate mental health care in the UK remains fragmented. This review evaluates the evidence for current UK neuropsychiatric service models and explores strategies to improve integration and access.

A targeted narrative review of MEDLINE, PsycINFO, and Embase (2000-2025) examined studies evaluating neuropsychiatric service models, including outcomes such as access, cost-effectiveness, patient outcomes, and clinician perspectives. Citation tracking was used to identify seminal models, including Sharpe’s integrated approach.

While liaison psychiatry services are present in over 90% of UK hospitals with emergency departments, many fall short of recommended staffing and capacity. Specialist inpatient neuropsychiatry units improve outcomes through multidisciplinary care but are geographically limited. Integrated neuropsychiatry services, such as the Oxford model, show the greatest clinical and economic promise by embedding psychiatry within neuroscience teams, though scalability is constrained by workforce shortages, fragmented commissioning, and inconsistent evaluation.

Integrated care offers the most effective means of addressing neuropsychiatric need. Achieving equitable access will require workforce expansion, cross-specialty training, unified funding mechanisms, and robust evaluation to ensure consistent, evidence-based care across the UK.

## Introduction and background

Neuropsychiatry occupies a critical yet often under-recognized position at the intersection of neurology and psychiatry. Its foundational aim - to address psychiatric syndromes that arise directly from brain dysfunction - is strongly supported by clinical evidence [1]. Psychiatric comorbidity is now understood to be a central feature of neurological disease: it reshapes clinical interpretation in epilepsy [2] and represents a major driver of disability in Parkinson’s disease [3]. Similarly, in multiple sclerosis, depression and anxiety are prevalent and intrinsic features of the condition, with pooled data demonstrating a substantial and enduring burden [4]. Despite this growing recognition, a significant and concerning gap remains between conceptual understanding and the delivery of care. Within the UK healthcare system, access to integrated, psychologically informed neuropsychiatric services is inconsistent and often inequitable, largely shaped by variable local commissioning priorities [5].

This substantial neuropsychiatric burden, however, remains consistently under-recognised and under-treated. In Parkinson’s disease, for example, depressive and anxiety symptoms frequently precede overt motor impairment, supporting a model of shared neurobiological vulnerability rather than purely reactive psychological distress [6]. Similarly, meta-analytic evidence confirms that mood and anxiety disorders affect a substantial proportion of multiple sclerosis patients across the entire disease course, with prevalence estimates commonly approaching half of all individuals [4]. The stakes are high: in epilepsy, suicide risk is significantly elevated compared with the general population [7,8].

Despite this clear evidence, UK patient-reported survey data demonstrate that many people living with neurological conditions do not receive timely or adequate mental health support, with clear downstream consequences for wellbeing [5]. Advocacy reports describe the cumulative harm of repeated dismissal or of being “unbelieved” - experiences that compound distress and actively delay engagement with care [9].

Huntington’s disease (HD) illustrates these systemic failures with particular starkness. Here, psychiatric symptoms such as depression, irritability, and apathy frequently precede a formal motor diagnosis by several years [10,11]. This creates a prolonged diagnostic limbo - a window where clinical need is substantial, yet care pathways remain poorly defined and access is obstructed. While studies meticulously document the timing and clinical impact of these symptoms [11], the patient experience reveals a persistent disconnect. Symptoms are too often framed as an “inevitable progression” of the disease rather than as treatable comorbidities, a fatalistic view that still frequently bars access to dedicated mental health support [9]. The consequence is a profound and preventable escalation of both suffering and clinical risk, highlighting a critical failure to translate robust clinical evidence into compassionate, effective care.

This reality of unmet need stems directly from entrenched structural divisions between neurology and psychiatry within healthcare systems. The problem is systemic. In the UK, the divide is codified early, through separate training paradigms: neurology curricula emphasise localisation and disease-focused management [12], while psychiatry training foregrounds syndromic diagnosis and bio-psychosocial formulation [13]. For patients whose conditions inherently span this artificial border, the system itself becomes a barrier to integrated care.

The clinical consequences are both predictable and severe. Depression is frequently under-recognised in non-psychiatric settings; a meta-analysis confirms that physicians in these contexts miss a substantial proportion of cases even when symptoms are evident [14]. In Parkinson’s disease, qualitative studies reveal that barriers to mental health support - including diagnostic overshadowing, stigma, uncertainty over clinical responsibility, and restrictive service criteria - persist despite well-documented need [15]. National patient survey data corroborates this, describing experiences of protracted waits, repeated referral loops between specialties, and the persistent framing of psychological care as ancillary to neurological management [5]. Collectively, these are inevitable failures of a siloed care model.

The experience of patients with functional neurological disorder (FND) offers a particularly stark illustration of these systemic shortcomings. FND is characterised by genuine, disabling neurological symptoms, such as weakness, tremor, or dissociative seizures, that arise from disturbances in nervous system activity [16]. While contemporary practice rightly advocates for a positive diagnosis based on specific clinical features, and despite robust evidence for targeted interventions (exemplified by the CODES trial, which demonstrated efficacy for specialised psychological therapy in dissociative seizures [17]), a critical implementation gap remains. All too often, patients receive a clear diagnosis only to encounter an ill-defined or non-existent care pathway, especially in regions without commissioned, multidisciplinary neurorehabilitation or specialist psychological services [5,18]. 

Syntheses of UK evidence consistently reveal ethnic inequalities in access to, experience of, and outcomes from mental healthcare [19,20]. These disparities are driven by discrimination, cultural insensitivity, and a deep-seated mistrust shaped by prior negative encounters with services [19,20]. For Black and minority ethnic communities, there are additional perceived barriers to engagement, including stigma, limited cultural responsiveness, and concerns about the acceptability of care [21]. Therefore, such layered challenges compound the existing difficulties of navigating fragmented neuropsychiatric pathways, creating a formidable, multi-layered barrier to appropriate support [22].

Large population-based studies underscore a stark reality: people with neurological disorders face a substantially elevated risk of suicide. One cohort study found the suicide rate among this population was 44.0 per 100,000 person-years, compared to 20.1 in the general population [23]. Condition-specific risks were even more alarming, with rates approaching a fivefold increase for diagnoses like HD and motor neurone disease [23]. These statistics point to periods of acute vulnerability - around diagnosis, progression, or healthcare contact - when integrated mental health support is often absent from standard neurological care.

In the UK context, the dominant national response to common mental health needs has been the Improving Access to Psychological Therapies (IAPT) programme, now known as NHS Talking Therapies. While evaluations show it has expanded access to treatment for depression and anxiety, it also highlights persistent challenges in equity and managing complexity [24]. For people with neurological conditions, engagement with IAPT is often constrained. Restrictive referral criteria, poor integration with specialist neurology, and limited adaptation for cognitive or physical comorbidities can render this primary-care model a poor fit, reinforcing the experience of psychological care as a parallel, disconnected service rather than an integrated component of holistic neurological management [22,24].

Therefore, despite broad consensus on the high prevalence and impact of psychiatric comorbidity in neurology, how UK services respond in practice remains unaddressed. This narrative review consequently aims to: (1) Examine contemporary UK service models that address mental health in neurological populations; (2) Identify the structural and organisational barriers that perpetuate fragmented care; and (3) Evaluate evidence-informed approaches that could forge stronger, more effective coordination between neurological and mental health services.

What neuropsychiatric service models exist in the UK?

In theory, the UK's mental healthcare system offers multiple tiers of support for people with neurological conditions, spanning primary care psychological services, secondary mental health care, and specialist tertiary provision. Yet in practice, the structure and remit of these tiers often create predictable gaps for patients whose psychiatric needs are inseparable from their neurological disease. The result is a landscape of fragmented and inconsistent care. Key features of this provision are summarised in Table 1.

**Table 1:** 2025 Snapshot of UK Neuropsychiatry Services Around the NHS N/A: not applicable; FND: functional nerve disorders

NHS TRUST	DEDICATED NEUROPSHYCIATRY TEAM?	IN-PATIENT SERVICES	SPECIALIST CLINIC/FOCUS	REFERENCE
South London and Maudsley	Yes	15 beds	FND, Acquired Brain Injury, Encaphalitis	[25]
Oxford Health	Yes	18 beds	Memory and Cognitive Assessment	[26]
St George’s (London)	Yes	Provides specialised liaison neuropsychiatry input (assessment, advice and time-limited management) to inpatients admitted to neurological, neurosurgical and neurocritical care beds on neurosciences wards at St George's Hospital.	FND, Epilepsy	[27]
Northern Care Alliance (Manchester Centre for Clinical Neurosciences)	Yes	Takes place at Salford Royal Foundation Trust. Specialist input is provided to the regional Huntington’s disease service at Manchester Royal infirmary and the David Lewis Centre for Epilepsy in Cheshire.	Acquired Brain Injury, Huntingtons Disease, Epilepsy, Parkinson’s	[28]
Leeds Teaching Hospitals	Yes	Provides inpatient neuropsychological assessment and therapeutic work to patients admitted to the neurorehabilitation ward at Chapel Allerton Hospital.	Medically Unexplained Symptoms	[29]
North Bristol NHS Trust	Yes	N/A	Stroke, Epilepsy, Multiple sclerosis, Cognitive disorders (dementia) clinic.	[30]
Birmingham and Solihull Mental Health Trust	Yes	N/A	Seizure disorders, including epilepsy with mental health co-morbidity and non epileptic attack disorder Sleep disorders, Tourette syndrome, Huntington’s disease, Chronic fatigue syndrome, Conversion disorders	[31]
University College London Hospitals	Yes	Inpatient admissions to Hughlings Jackson ward, a tertiary referral unit. Consultation-liaison neuropsychiatric service for inpatients admitted to neurological, neurosurgical and neuro critical care beds at NHNN.	Epilepsy, Parkinson’s, Multiple sclerosis, Encephalitis	[32]
North Staffordshire Combined Healthcare	Yes	N/A	Acquired Brain Injury	[33]
Avon and Wiltshire Mental Health Partnership	No	Complete gatekeeping assessments for people brain injuries who present a risk to others and may need admission to secure services. Provides an outreach and liaison service to support individuals leaving hospital to return to the community.	Brain Injuries	[34]
Sheffield Teaching Hospitals	No	Referred by Neurology Department in Sheffield Teaching Hospital	FND, Epilepsy	[35]

Primary Care Psychological Therapies (IAPT/NHS Talking Therapies)

At the frontline, the IAPT programme provides a national framework of evidence-based, short-term psychological interventions, predominantly cognitive behavioural therapy for mild-to-moderate depression and anxiety [24]. Operating mainly within primary care, these services rely on stepped-care models and protocol-driven treatments, accessible via GP or self-referral.

National guidance explicitly acknowledges the need to adapt these therapies for people with long-term physical health conditions. Recommended adjustments include flexible pacing, altered communication styles, and accommodations for cognitive or physical limitations [22]. The intent is to widen access.

However, the very features that allow IAPT to function at scale - standardised pathways, session limits, and strict eligibility - often render it a poor fit for neurological complexity [24]. Patients may present with fluctuating symptoms, cognitive impairment, or diagnostic ambiguity, which sit uneasily within brief, manualised treatments. Consequently, individuals with progressive or complex neurological disorders are frequently triaged as unsuitable for IAPT, despite significant psychological distress [22,24]. While the service may help some, it systematically struggles to accommodate the layered and evolving reality of neuropsychiatric illness.

Secondary Mental Health Care (Community Mental Health Teams, Crisis Teams, Inpatient Psychiatry)

The next tier comprises Community Mental Health Teams (CMHTs), crisis resolution services, and inpatient psychiatry. These services focus on severe, enduring, or high-risk mental illness, with access governed by diagnostic categories and perceived clinical risk [15-18]. 

For neurological patients, the central difficulty is one of clinical ‘fit.’ Psychiatric symptoms may be severe but atypical, explicitly linked to brain disease, or complicated by cognitive or functional deficits [16,18]. Such presentations often defy psychiatric categorisation, creating confusion over triage and clinical responsibility. Research into conditions like FND illustrates this well: patients frequently oscillate between neurology and psychiatry, with neither service assuming sustained ownership, leading to cycles of reassessment rather than coherent treatment [16,18].

The human impact of this ambiguity is clear. In Parkinson’s disease, patients report diagnostic overshadowing, stigma, and service thresholds that block access to mental health support, despite high levels of need [15]. Similarly, in HD, advocacy reports describe how symptoms are often dismissed as an inevitable part of neurological decline, rather than treatable conditions, leading to harmful delays and repeated dismissal [9]. These experiences are symptomatic of a system ill-equipped to manage problems that straddle its foundational divisions.

Across policy, clinical guidance, and research, a consensus is clear: psychiatric comorbidity in neurological disease is not just common, it is a central driver of poor outcomes. The evidence is compelling. Cohort studies show that psychiatric symptoms often herald or accompany a neurological diagnosis, underscoring the critical need for proactive, integrated mental healthcare, not just reactive crisis management [10,11]. This principle is even embedded in national guidance for psychological therapies, which explicitly calls for adapted, not excluded, care for people with long-term physical health conditions [22].

Yet, as the previous sections have detailed, this consensus fractures upon contact with the reality of service provision. Both primary care psychological services (IAPT/NHS Talking Therapies) and secondary mental health care are structurally constrained - bound by eligibility thresholds, narrow diagnostic categories, and rigid service remits. Consequently, for many patients, care is not integrated but fragmented: delivered in disconnected parallel streams across different settings, or not delivered at all. The system recognises the need but remains architecturally incapable of fulfilling it.

Liaison Neuropsychiatry Services

Liaison psychiatry services occupy a critical and often overburdened interface between acute medicine, mental health services, and community care. They are frequently positioned as the compensatory mechanism of last resort when primary and secondary mental health pathways prove unable to accommodate neuropsychiatric complexity [36,37].

The Fourth Annual Survey of Liaison Psychiatry found that while most hospitals with emergency departments had some access to liaison psychiatry, only a minority met recommended staffing standards, and maintaining rapid response times remained a widespread challenge [36]. Complementary national surveys corroborate this picture of broad but inconsistent geographic coverage [37].

Where robustly implemented, the value of liaison psychiatry is undeniable, particularly within the acute hospital setting. The pivotal HOME Study, a multicentre randomised controlled trial, demonstrated that proactive, consultant-led liaison psychiatry for older medical inpatients significantly reduced their length of hospital stay compared to standard care [38]. Qualitative evaluations reinforce this, with referring clinicians reporting improved decision-making and management planning, and nursing staff emphasising the crucial support provided during acute crises [39].

The economic case is also compelling. An evaluation of the Birmingham Rapid Assessment Interface and Discharge (RAID) model showed that effective liaison psychiatry reduced both length of stay and readmission rates, generating substantial bed-day savings for acute trusts [40]. Furthermore, extended liaison service hours are associated with better psychosocial assessments after self-harm and a reduction in patients discharging themselves prematurely [40].

Yet the very position of liaison psychiatry within the care pathway also exposes its core limitation. These services are fundamentally designed for acute episodes, crisis management, and in-hospital risk. For patients with chronic, progressive neurological conditions, liaison input may resolve an immediate emergency but rarely provides the continuity of psychiatric care required once the acute phase passes. This creates a recurrent cycle, vividly illustrated in studies of FND, where patients move between neurology, liaison psychiatry, and community services without any service assuming sustained responsibility [16,18]. Disease-specific evidence and patient testimony echo this pattern, describing how it leads to repeated reassessment, delayed treatment, and persistently unmet needs in conditions like Parkinson’s and Huntington’s disease [9,15].

Inpatient Neuropsychiatry Units

At the tertiary level of care, specialist inpatient neuropsychiatry units treat patients with the most severe and complex needs. These patients typically have serious neurological conditions alongside psychiatric, cognitive, or behavioural difficulties. Their care cannot be safely or effectively provided in standard medical or general psychiatric wards [41].

Evidence from service evaluations and commissioning analyses indicates that insufficient specialist neuropsychiatric capacity generates system-wide inefficiencies, including prolonged hospital admissions, delayed discharges, and impaired patient flow across acute and mental health services [42,43]. In this context, fragmentation between neurology and psychiatry is a driver of avoidable clinical and operational harm.

The core value proposition of inpatient neuropsychiatry units lies in integration. These services provide a consolidated alternative to the fragmented and often damaging pathway of separate neurology and psychiatry admissions by delivering coordinated neurological assessment, specialist psychiatric care, and intensive psychological intervention within a single multidisciplinary team. This model aligns with evidence from consultation-liaison psychiatry, which demonstrates improvements in care coordination and professional satisfaction, although effects on long-term outcomes are mixed [44].

Despite this clear rationale, national provision remains insufficient and uneven. Service mapping studies reveal marked geographic variability, with specialist units concentrated in a small number of centres and referral rates far below estimated population need [41]. Structural barriers - including fragmented funding streams, limited commissioning expertise, and the absence of clear national service specifications - continue to impede service development [42,43].

The downstream consequences of constrained capacity are severe and visible. National data show that shortages in specialist mental health beds routinely result in distressing out-of-area placements. In 2023 alone, more than 5,000 patients in England were treated over 100 km from home because appropriate local beds were unavailable [45]. Although this figure spans all mental health services, it starkly illustrates the systemic crisis created by inadequate specialist provision.

In summary, inpatient neuropsychiatry units fulfil a critical role for a highly vulnerable patient group, yet current provision remains a niche resource that falls well short of demand. Addressing this gap requires strategic expansion of specialist capacity, underpinned by clearer commissioning frameworks and deliberate integration with community and acute services [41-46].

Integrated Neuropsychiatry Services

The Oxford Integrated Neuropsychology and Neuropsychiatry Service exemplifies the United Kingdom’s most advanced approach to integrated care for patients whose conditions bridge neurological and psychiatric presentations. By uniting specialists from neurology, psychiatry, clinical neuropsychology, and rehabilitation, the service enables truly collaborative assessment and formulation for individuals whose symptoms cut across conventional diagnostic categories [47].

A distinctive strength of the Oxford model lies in its deep academic-clinical partnership with the University of Oxford. This collaboration fosters a two-way exchange: complex clinical cases inform research agendas, while new scientific insights are rapidly incorporated into practice. The university setting also cultivates essential specialist training in neuropsychiatry and neuropsychology, helping to build a sustainable workforce equipped to manage complex presentations [48]. In practice, the service supports a highly heterogeneous caseload, including patients with functional neurological disorder, neuropsychiatric symptoms of dementia, epilepsy, and dissociative seizures, reflecting the wide range of needs that integrated care models are designed to address [47].

Nevertheless, the concentration of such services within major academic centres points to systemic barriers that have hindered broader adoption. National policy analyses consistently point to fragmented commissioning as a key obstacle, with neuropsychiatry often falling ambiguously between neurological and mental health funding streams. This leads to unclear accountability and limited resources [49]. These structural challenges are compounded by workforce shortages and uneven access to specialist training, effectively confining comprehensive neuropsychiatry services to a few well-resourced regions [48,49].

Broader health system reviews reinforce this analysis, highlighting how organisational fragmentation and weak integration across health and social care undermine coherent care pathways for patients with multi-domain needs [49]. While centres such as Oxford can overcome these barriers locally - leveraging robust academic infrastructure - most regions lack the necessary conditions to replicate such models.

Thus, the Oxford service serves both as a proof of concept and a revealing case study of the systemic constraints facing UK neuropsychiatry. The central challenge is no longer proving clinical value, but rather reforming commissioning structures, expanding workforce capabilities, and nurturing academic-clinical partnerships so that integrated neuropsychiatry can be delivered sustainably and equitably at a national scale [49].

## Review

Methods

Search Strategy

This narrative review employed a targeted search strategy to evaluate neuropsychiatry service models across four key domains: (1) cost-effectiveness, (2) patient outcomes, (3) service access, and (4) clinician perspectives. The search encompassed three major databases (MEDLINE, PsycINFO, and Embase) for peer-reviewed articles published between January 2000 and April 2025.

The search syntax combined three concept clusters using Boolean operators:

Service model terms: ("neuropsychiatr* service* model" OR "integrated neuropsychiatr" OR "liaison psychiatr*")

Population terms: ("neurolog" OR "neuropsychiatr disorder*")

Outcome terms: ("cost-effectiveness" OR "patient outcome" OR "service access" OR "clinician perspective")

Inclusion and Exclusion Criteria

Filters were applied for English language and human studies. Recognizing that some seminal works might not appear in database searches, supplementary methods were employed to identify key publications. This included citation tracking of influential papers and targeted inclusion of Michael Sharpe's works on consultation-liaison psychiatry models [39], which represent contributions to the field despite not being captured through standard database searches. Table 2 depicts the inclusion and exclusion criteria used to screen studies.

**Table 2:** Inclusion and Exclusion Criteria

Category	Inclusion Criteria	Exclusion Criteria
Study Design	Primary studies evaluating service models (RCTs, cohort, case-control, service evaluations)	Commentaries, editorials, protocols without outcome data
Population	Adults (≥18y) with neurological conditions and psychiatric comorbidities	Paediatric-only populations
Intervention	Defined neuropsychiatry service models (liaison, integrated, inpatient)	Pharmacological studies without service delivery analysis
Outcomes	Quantitative data on cost, clinical outcomes, access metrics, or clinician views	Qualitative-only studies without measurable outcomes
Setting	High-income countries (prioritizing UK/NHS where available)	Low-resource settings without model generalizability

Selection of Studies

**Figure 1:**
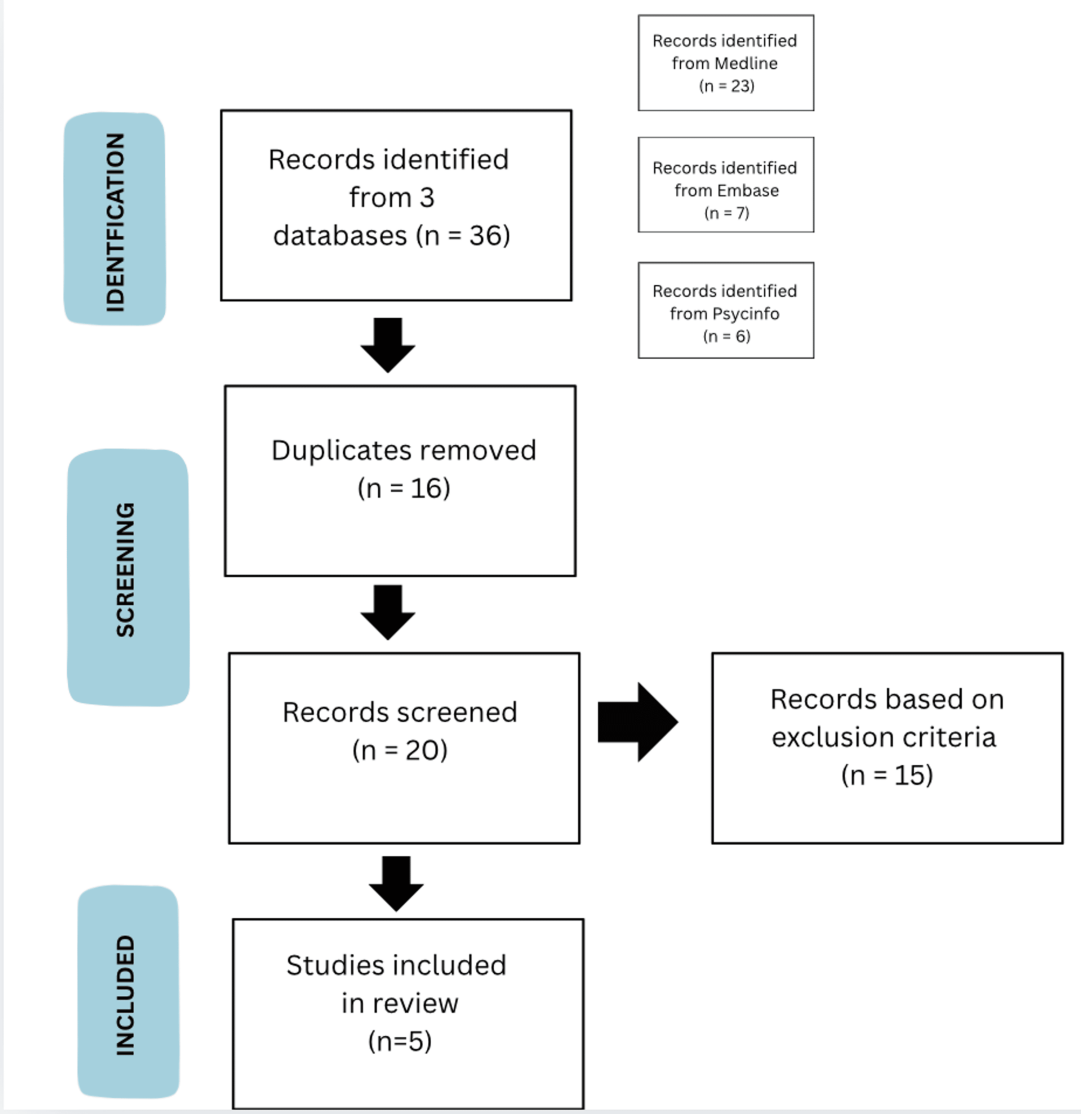
Preferred Reporting Items for Systematic Reviews and Meta-Analyses (PRISMA) Diagram

After removal of duplicates, titles and abstracts were screened for relevance. Full texts of potentially eligible articles were then reviewed against inclusion criteria. The Preferred Reporting Items for Systematic Reviews and Meta-Analyses (PRISMA) flow diagram (Figure 1) documents this process. 

Results

The evidence supporting integrated neuropsychiatric care models presents a paradox of promise and limitation, as demonstrated by Table 3.

**Table 3:** Summary of Key Studies Included in Review RCT: randomized controlled trials, CBT: Cognitive behavioral therapy, ILAE: International League Against Epilepsy

Authors	Study	Study Type	Population	Intervention / Focus	Key Findings	Reference
Sharpe M, Walker J, van Niekerk M, Toynbee M, Magill N, Frost C, White IR, et al.	Proactive integrated consultation-liaison psychiatry and time spent in hospital by older medical inpatients in England (The HOME Study)	Multicentre, parallel-group randomised controlled trial	2744 older inpatients (1399 male [51%]; 1345 female [49%])	Proactive Integrated Consultation-Liaison Psychiatry (PICLP) vs usual care	PICLP did not significantly reduce total time in hospital compared with usual care; process and qualitative evaluations indicate improved care coordination and discharge planning	[38]
Gandy M, Michaelis R, Acraman J, Donald KA, Fitzpatrick M, LaFrance WC Jr, et al.	Integrated psychological care services within seizure settings: Key components and implementation factors among example services in four ILAE regions	Descriptive service report (ILAE Psychiatry Commission)	Eight seizure care services (three pediatric, five adult); sex distribution not reported in service descriptions	Description of integrated psychological care service models for epilepsy/seizure clinics	Identified core operations, implementation factors, and practical tips for building integrated psychological care services; future work needed to systematically examine clinical impact and cost-effectiveness	[50]
Goldstein LH, Robinson EJ, Pilecka I, et al.	Cognitive-behavioural therapy compared with standardised medical care for adults with dissociative non-epileptic seizures (CODES RCT)	Pragmatic multicentre, parallel-arm randomised controlled trial	368 adults with dissociative seizures (sex breakdown not routinely included in abstract; likely near balanced but need to derive)	12-session dissociative seizure-specific CBT + standardised medical care vs standardised medical care alone	CBT plus standardised care did not significantly reduce seizure frequency over 12 months compared with medical care alone but was associated with benefits on some secondary outcomes (psychosocial functioning, periods free of seizures, satisfaction); cost-effectiveness was low	[17]
Russo G, Jia L, Kim CY, Stojanovic K, Wesley SF, Denfield GH, Askalsky P, Noy G, Thakur KT	Developing a clinical workflow for early recognition and diagnosis of autoimmune encephalitis in patients presenting with atypical psychosis	Clinical workflow development / service innovation study	Not a patient cohort study; illustrative clinical cases only (sex distribution not applicable)	Development of a structured diagnostic workflow to support early identification of autoimmune encephalitis in patients presenting with atypical psychosis in psychiatric and general hospital settings	The proposed workflow highlights clinical “red flags,” recommends early neurological consultation and antibody testing, and aims to reduce diagnostic delay and misdiagnosis of autoimmune encephalitis as primary psychiatric illness	[51]
Thyrian JR, Hertel J, Wucherer D, et al.	Implementing Dementia Care Management into routine care: protocol for a cohort study in Siegen-Wittgenstein, Germany (RoutineDeCM)	Cohort study protocol	Protocol specifies people with dementia in routine care; sex breakdown will be reported in results publications	Implementation of dementia care management within routine care	Study protocol outlining methods and expected outcomes for evaluating routine dementia care management implementation	[52]

The most robust evidence to date comes from the HOME Study, a multicentre randomised controlled trial of a Proactive Integrated Consultation-Liaison Psychiatry (PICLP) model delivered to 2,744 older medical inpatients [38]. Although the intervention did not significantly shorten total hospital stay compared with usual care, it produced meaningful improvements in care processes: psychiatric assessments were completed more quickly, coordination between medical and mental health teams strengthened, and discharge planning became more structured. Qualitative findings also indicated high levels of acceptance among both patients and staff [38].

Beyond general hospital liaison psychiatry, the evidence remains more developmental. Russo et al., for example, proposed a structured clinical workflow to aid early recognition of autoimmune encephalitis in patients presenting with atypical psychosis, incorporating defined clinical “red flags” and prompt neurological consultation [51]. This approach represents a valuable step toward standardising diagnosis and refining service pathways, though it does not yet report data on patient outcomes or comparative effectiveness [51].

In a similar vein, the International League Against Epilepsy (ILAE) Psychiatry Commission report described eight integrated psychological care services embedded in epilepsy clinics across four international regions [50]. These exemplar programmes commonly featured close collaboration between neurologists and psychologists, along with flexible referral routes. However, the absence of comparative effectiveness or economic data limits what can be concluded about their clinical impact [50].

The CODES trial offers the strongest evidence for a targeted psychological intervention in a neuropsychiatric-adjacent population. In this pragmatic randomised controlled trial involving 368 adults with dissociative seizures, cognitive-behavioural therapy combined with standardised medical care did not achieve a statistically significant reduction in seizure frequency at 12 months, compared with medical care alone [17]. Still, several secondary outcomes, e.g., psychosocial functioning, seizure-free intervals, and patient satisfaction favoured the intervention, albeit with accompanying economic analyses suggesting limited cost-effectiveness [17].

Finally, evidence within neurodegenerative disease remains preliminary. The RoutineDeCM study, currently outlined in a published protocol, describes a structured biopsychosocial dementia care-management intervention with planned recruitment of 90 participants [52]. As a protocol paper, it contributes to implementation planning but does not yet offer evidence on clinical or cost-effectiveness.

Discussion

The findings of this review echo enduring themes in the literature on integrated psychiatric care, while also underscoring the field's persistent methodological constraints. A recurring pattern emerges: interventions often enhance care processes without reliably improving measurable utilisation outcomes. 

The HOME Study's null primary outcome showing no significant reduction in length of stay despite better clinical coordination contrasts with earlier, often less rigorous, evaluations [38]. Previous national surveys and service reviews consistently reported high clinician satisfaction and perceived improvements in care quality linked to liaison psychiatry models [36,37,41,42], while economic analyses, such as those of the RAID model, projected considerable savings [40]. The more rigorous design of the HOME Study suggests these earlier estimates may have been overly optimistic, offering a tempered view of how liaison services affect hard system metrics. This does not devalue improvements in coordination, but clarifies that such benefits may not translate into short-term reductions in hospital bed use.

Where the HOME Study advances the field is in demonstrating scalability, proving a proactive liaison model can be implemented across multiple NHS sites [38]. This supports prior advocacy for proactive approaches, while also highlighting the ongoing constraint of specialist workforce capacity, a limitation repeatedly noted in national service analyses [36,41,42].

In neuropsychiatric diagnostics, the clinical workflow proposed by Russo et al. responds directly to well-recognised problems of diagnostic delay and misattribution in psychiatric settings [51]. Its contribution is conceptual, aligning with expert consensus on the need for structured pathways. Yet, like much of the literature in this area, it illustrates a broader tendency to advance service innovations based on clinical logic and case evidence, without the comparative outcome data needed to establish effectiveness. This reflects a wider pattern in neuropsychiatry of prioritising service development over controlled evaluation.

The descriptive findings of the Psychiatry Commission report align with long-standing international calls for integrated psychological care in epilepsy [50]. Although such embedded services are widely promoted to improve access and continuity, the continued lack of standardised evaluation means the evidence remains rich in description but poor in comparative effectiveness data [2,50]. Integration is frequently recommended, yet its impact on key patient outcomes remains inadequately demonstrated.

The mixed results of the CODES trial reflect the inherent complexities of treating functional disorders [17]. A null finding on the primary outcome (seizure frequency) coexisted with improvements in secondary measures like functioning and satisfaction - a pattern seen in earlier psychological intervention trials [16,17]. This reinforces the argument that symptom frequency alone is an insufficient measure of success. While CODES marks a methodological advance in scale and transparency, its findings underscore the challenge of defining outcomes that matter both to patients and to healthcare systems [16,17].

In neurodegenerative disease, the reliance on protocol papers such as that for RoutineDeCM indicates the evidence base is still formative [52]. This mirrors the broader trajectory of dementia-care research, where promising models often undergo extended feasibility testing, delaying robust conclusions about clinical and cost-effectiveness.

Taken together, the heterogeneous outcomes, scarce economic evaluation, and inconsistent demographic reporting, are not new [36,41,42]. Their persistence across decades points to structural challenges in evaluating complex system-wide interventions, rather than mere methodological oversights. This stands in stark contrast to consistent testimony from patient organisations documenting the real harms of fragmented care and unmet need [5,9].

Recommendations and future directions

The evidence reviewed points to several priorities for advancing neuropsychiatric care. First, there is a clear need to integrate psychiatric expertise more deeply within neurology and neuroscience services. Models that embed psychiatrists as core members of multidisciplinary teams show promise for improving diagnostic accuracy and care continuity [35]. Achieving this, however, will require confronting systemic barriers, especially the fragmented commissioning and organisational silos that still hinder collaborative practice.

Alongside structural reform, expanding neuropsychiatric training for both neurologists and psychiatrists is essential. Current educational pathways often leave clinicians underprepared for the high prevalence of conditions that straddle neurological and psychiatric domains. Reforming curricula through mandatory neuropsychiatry exposure and the development of dual-competency training pathways would better equip the workforce to manage complex, cross-boundary presentations.

Finally, future research must commit to the rigorous, systematic evaluation of integrated service models. Promising embedded approaches should be tested using comparative designs and standardised outcome measures that capture not only clinical endpoints, but also patient-reported experience and functioning. Special focus should be given to improving access for neurological patients who are often excluded from mainstream mental health services due to physical or cognitive complexity.

By addressing these clinical, educational, and systemic gaps in a coordinated manner, the field can move toward a more sustainable and equitable standard of neuropsychiatric care.

## Conclusions

This review underscores a deeply human problem within contemporary healthcare systems: individuals living with neurological conditions too often navigate their psychological distress alone, despite a high prevalence of co-occurring psychiatric illnesses. The enduring separation between neurological and psychiatric services fragments the person at the very moment they require holistic understanding, creating structural barriers to compassionate, comprehensive care. Compelling evidence now affirms that integrated care models - where neurologists and psychiatrists collaborate as a unified team around the patient - restore coherence to the clinical encounter. Such models enhance diagnostic clarity, unify treatment plans, and provide the continuity of care necessary to address the intertwined nature of brain and mind.

Translating this evidence into practice demands deliberate, system-wide evolution. This includes cultivating a shared foundation of neuropsychiatric knowledge among clinicians, redesigning care pathways to foster authentic collaboration, and reforming funding mechanisms to reward integrated care rather than siloed interventions. The undertaking is considerable, yet it holds the promise of care that is not only more effective and efficient but also more humane. Future efforts must prioritize the meticulous measurement of patient outcomes and experiences, ensuring that the voices of those served guide the process. Additionally, detailed documentation of implementation successes and challenges will be crucial for scaling these models. The accumulating evidence positions integrated care not merely as a logistical option, but as an ethical and practical imperative for a healthcare system aspiring to treat the whole person.

## References

[1] (2013). Behavioral Neurology & Neuropsychiatry. https://books.google.co.uk/books?hl=en&lr=&id=GvvDpw-oCWIC&oi=fnd&pg=PR8&dq=Arciniegas+DB,+Anderson+CA,+Filley+CM,+editors.%C2%A0Behavioral+neurology+%26+neuropsychiatry.%C2%A0Cambridge:+Cambridge+University+Press%3B+2013.&ots=4Csa5JScLa&sig=DagpMAEBktMUdjxRvBEPmpZ5Q78&redir_esc=y#v=onepage&q&f=false.

[2] Kanner AM (2016). Psychiatric comorbidities in epilepsy: should they be considered in the classification of epileptic disorders?. Epilepsy Behav.

[3] Weintraub D, Aarsland D, Chaudhuri KR (2022). The neuropsychiatry of Parkinson’s disease: advances and challenges. Lancet Neurol.

[4] Boeschoten RE, Braamse AMJ, Beekman ATF (2017). Prevalence of depression and anxiety in multiple sclerosis: a systematic review and meta-analysis. J Neurol Sci.

[5] Neurological Alliance. Together for the 1 in 6 (2026). Together for the 1 in 6: UK findings from My Neuro Survey. https://www.neural.org.uk/togetherforthe1in6/.

[6] Jacob EL, Gatto NM, Thompson A, Bordelon Y, Ritz B (2010). Occurrence of depression and anxiety prior to Parkinson’s disease. Parkinsonism Relat Disord.

[7] Christensen J, Vestergaard M, Mortensen PB, Sidenius P, Agerbo E (2007). Epilepsy and risk of suicide: a population-based case-control study. Lancet Neurol.

[8] Abraham N, Tran BX, Ho RCM (2019). A meta-analysis of suicide ideation, attempts and deaths in people with epilepsy. Int J Environ Res Public Health.

[9] (2026). Unseen and unheard: the need to improve mental healthcare for people living with Huntington’s disease. https://www.hda.org.uk/news/unseen-and-unheard-mental-health-in-huntingtons-disease/.

[10] Julien CL, Thompson JC, Wild S (2007). Psychiatric disorders in preclinical Huntington’s disease. J Neurol Neurosurg Psychiatry.

[11] McAllister B, Gusella JF, Landwehrmeyer GB (2021). Timing and impact of psychiatric, cognitive, and motor abnormalities in Huntington disease. Neurology.

[12] (2019). Adams and Victor's Principles of Neurology.

[13] Engel GL (1977). The need for a new medical model: a challenge for biomedicine. Science.

[14] Cepoiu M, McCusker J, Cole MG (2008). Recognition of depression by non-psychiatric physicians: a systematic literature review and meta-analysis. J Gen Intern Med.

[15] Dobkin RD, Rubino JT, Friedman J (2013). Barriers to mental health care utilization in Parkinson’s disease. J Geriatr Psychiatry Neurol.

[16] Espay AJ, Aybek S, Carson A (2018). Current concepts in diagnosis and treatment of functional neurological disorders. JAMA Neurol.

[17] Goldstein LH, Robinson EJ, Pilecka I (2021). Cognitive-behavioural therapy compared with standardised medical care for adults with dissociative non-epileptic seizures: the CODES RCT. Health Technol Assess.

[18] Stone J, Carson A, Duncan R (2009). Symptoms “unexplained by organic disease” in 1144 new neurology out-patients: how often does the diagnosis change at follow-up?. Brain.

[19] Bansal N, Karlsen S, Sashidharan SP (2022). Understanding ethnic inequalities in mental healthcare in the UK: a meta-ethnography. PLoS Med.

[20] Nazroo JY, Bhui KS, Rhodes J (2020). Where next for understanding race/ethnic inequalities in severe mental illness? Structural, interpersonal and institutional racism. Sociol Health Illn.

[21] Memon A, Taylor K, Mohebati LM (2016). Perceived barriers to accessing mental health services among Black and minority ethnic communities: a qualitative study. BMJ Open.

[22] (2018). The IAPT Pathway for People With Long-Term Physical Health Conditions and Medically Unexplained Symptoms: Full Implementation Guidance. NCCMH.

[23] Erlangsen A, Stenager E, Conwell Y (2020). Association between neurological disorders and death by suicide in Denmark. JAMA.

[24] Wakefield S, Kellett S, Simmonds-Buckley M (2021). Improving Access to Psychological Therapies (IAPT) in the United Kingdom: a systematic review and meta-analysis of 10 years of practice-based evidence. Br J Clin Psychol.

[25] (2026). South London and Maudsley. Neuropsychiatry services. Neuropsychiatry Services [Internet]. Available from.

[26] (2026). Oxford Health NHS Foundation Trust. Memory services. https://oxfordhealth.nhs.uk/service_description/memory-services/.

[27] (2026). South West London and St George’s Mental Health NHS Trust. Neuropsychiatry service. Neuropsychiatry Service [Internet.

[28] (2026). Manchester Centre for Clinical Neurosciences (MCCN). https://www.northerncarealliance.nhs.uk/our-services/mccn.

[29] (2026). Leeds Teaching Hospitals NHS Trust. Neuropsychology. https://www.leedsth.nhs.uk/services/clinical-and-health-psychology/neuropsychology/.

[30] (2026). North Bristol NHS Trust. Neuropsychology. Neuropsychiatry [Internet]. Available.

[31] (2026). Birmingham and Solihull Mental Health NHS Foundation Trust. Neuropsychiatry. Neuropsychiatry [Internet]. Available.

[32] (2026). University College London Hospitals NHS Foundation Trust. Neuropsychiatry. Neuropsychiatry [Internet]. Available.

[33] (2026). North Staffordshire Combined Healthcare NHS Trust. Neuropsychiatry community service. https://www.combined.nhs.uk/services/neuropsychiatry-community-service/.

[34] (2026). Avon and Wiltshire Mental Health Partnership NHS Trust. https://www.awp.nhs.uk.

[35] (2026). Sheffield Teaching Hospitals NHS Foundation Trust. Neuropsychology. https://www.sth.nhs.uk/patients-visitor-information/patient-information-leaflets/view-all-by-specialty/?i=Neuropsychology.

[36] (2026). Report of the 4th survey of liaison psychiatry in England (LPSE-4). https://www.england.nhs.uk/wp-content/uploads/2019/07/fourth-annual-survey-liaison-psychiatry-england-report.pdf.

[37] Walker A, Barrett JR, Lee W (2018). Organisation and delivery of liaison psychiatry services in general hospitals in England: results of a national survey. BMJ Open.

[38] Sharpe M, Walker J, van Niekerk M (2024). Proactive integrated consultation-liaison psychiatry and time spent in hospital by older medical inpatients in England (HOME Study): a multicentre, parallel-group, randomised controlled trial. Lancet Psychiatry.

[39] Solomons LC, Thachil A, Burgess C (2011). Quality of psychiatric care in the general hospital: referrer perceptions of an inpatient liaison psychiatry service. Gen Hosp Psychiatry.

[40] Tadros G, Salama R, Kingston P (2013). Impact of an integrated rapid response psychiatric liaison team on quality improvement and cost savings: the Birmingham RAID model. Psychiatrist.

[41] Agrawal N, Bhattacharya R, Rickards H (2015). Provision of neuropsychiatry services: variability and unmet need. BJPsych Bull.

[42] Bhattacharya R, Rickards H, Agrawal N (2015). Commissioning neuropsychiatry services: barriers and lessons. BJPsych Bull.

[43] Parsonage M, Fossey M (2011). Economic Evaluation of a Liaison Psychiatry Service. Health.

[44] Toynbee M, Walker J, Clay F (2021). The effectiveness of inpatient consultation-liaison psychiatry service models: a systematic review of randomised trials. Gen Hosp Psychiatry.

[45] (2023). No end in sight: over 5,000 cases of patients with mental illness sent more than 100 km away for treatment. https://www.rcpsych.ac.uk/news-and-features/latest-news/detail/2023/06/21/no-end-in-sight-over-5-000-cases-of-patients-with-mental-illness-being-sent-more-than-100km-away-for-vital-treatment.

[46] Opmeer BC, Hollingworth W, Marques EMR (2017). Extending the liaison psychiatry service in a large hospital in the UK: a before and after evaluation of the economic impact and patient care following emergency department attendances for self-harm. BMJ Open.

[47] (2026). Oxford Integrated Neuropsychology and Neuropsychiatry Service. https://oxfordhealth.nhs.uk/camhs/oxon/neuropsychiatry/.

[48] (2026). About the Department: research, clinical integration and training. https://www.psych.ox.ac.uk/about.

[49] (2026). Integrating health and social care: Sixtieth Report of Session 2016-17. https://publications.parliament.uk/pa/cm201617/cmselect/cmpubacc/959/959.pdf.

[50] Gandy M, Michaelis R, Acraman J (2023). Integrated psychological care services within seizure settings: key components and implementation factors among example services in four ILAE regions. Epilepsia.

[51] Russo G, Jia L, Kim CY (2025). Developing a clinical workflow for early recognition and diagnosis of autoimmune encephalitis in patients presenting with atypical psychosis. J Acad Consult Liaison Psychiatry.

[52] Thyrian JR, Hertel J, Wucherer D (2024). Implementing dementia care management into routine care: protocol for a cohort study in Siegen-Wittgenstein, Germany (RoutineDeCM). BMJ Open.

